# New Surgical Appliances

**Published:** 1889-02-23

**Authors:** 


### New Surgical Appliances.

How fortunate are the patients of to-day compared with those of fifty years ago! It is not merely in the greater matters of anæsthetics and manipulative dexterity, but in what may seem the smaller details of dressings and wound paddings also that there has been an advance which is as gratifying as it is creditable and remarkable. The Liverpool Patent Lint Company have achieved an honourable distinction in the excellence of a great variety of specialities. Their small box of six or eight samples of "wound padding," "splint padding," "proofed lint," "absorbent proofed felt," and a "health chest protector," cannot but gladden the heart of a surgeon who has a just appreciation of the new surgery, and any regard for the progress and comfort of his patients. The "splint padding" is admirable, and supplies a want which ordinary practitioners, at any rate, have often experienced. Using it there is hardly a possibility of any want of perfect apposition of parts in ordinary fractures. Of equal excellence and utility is the "complete surgical dressing." For cleanliness, comfort, and neatness it leaves nothing to be desired. The "wound padding" will make neat-handed clinical clerks, dressers and nurses quite happy, and will give as much satisfaction to the patient as it does to the attendant. It is not necessary to particularise in more detail these beautiful and useful surgical accessories. It may be said with truth that the whole series are excellent throughout.

